# Mechanisms of recovery: Community perceptions of change and growth following multiple disasters

**DOI:** 10.3389/fpsyg.2022.991770

**Published:** 2022-11-21

**Authors:** Howard Osofsky, Joy Osofsky, Leia Y. Saltzman, Estilla Lightfoot, Jule De King, Tonya C. Hansel

**Affiliations:** ^1^LSU Health Sciences Center New Orleans, Louisiana State University, New Orleans, LA, United States; ^2^Tulane School of Social Work, Tulane University, New Orleans, LA, United States; ^3^Western New Mexico University, Silver City, NM, United States

**Keywords:** PTG, community, oil spill, qualitative, Hurricane Katrina

## Abstract

**Introduction and purpose:**

The geographic location of the Gulf South leaves communities in continuous threat, response, and recovery disaster cycles. Hurricane Katrina in 2005 provided an opportunity to study disaster mental health. Less than 5 years after the storm, many Hurricane Katrina survivors were impacted again by the Deepwater Horizon Gulf oil spill. Despite adversities impacting Gulf communities, over 90% of participants reported they were resilient. The purpose of this study was to improve the understanding of the mechanisms that contribute to strengths following adversity in communities affected by repeated disasters. Specifically, we focused on survivor perceptions of personal, spiritual, or community changes in efforts to describe community resilience and posttraumatic growth (PTG).

**Methods:**

Participants were recruited through a quantitative survey and community flyers. Participants represented southeastern Louisiana, in areas impacted by hurricanes and the oil spill—for a total of five focus groups and 41 participants. Focus groups began by asking each participant to provide a brief overview of their disaster survival story and three additional guiding strengths-based questions. Data were transcribed using Dragon Speech Recognition software. A total of 963 unique responses were analyzed and coded.

**Results:**

The following themes were identified: connectedness (*n* = 259), coping (*n* = 94), spirituality (*n* = 60), adaptability (*n* = 47), and self-reliance (*n* = 23). Participants noted a growth mindset from the disasters and also acknowledged coinciding negative experiences (*n* = 154) associated with community change and loss, where subthemes included change in connectedness (*n* = 97), crime (*n* = 26), and feeling like an outsider (*n* = 31).

**Discussion and implications:**

These findings help scholars and mental health practitioners better understand the lived experiences of PTG in a community of survivors impacted by recurring traumatic experiences. In keeping with previous literature, PTG and negative experiences associated with trauma are not mutually exclusive, but occur simultaneously. Our results offer a holistic picture of coping with cumulative or repeated traumas and suggest that connectedness, coping, and spirituality provide important buffers to negative psychosocial outcomes.

## Introduction

The geographic location of the Gulf South exposes leaves communities to continuous threat, response, and recovery disaster cycles. Multiple and long-term disasters can negatively affect behavioral health, with the extensive literature noting the association among cumulative adversities and poor mental and physical health outcomes (Felitti, 2002; Shrira et al., 2010; Finkelhor et al., 2013; Lowe et al., 2013). Similar patterns of mental health concerns, for example, increased anxiety, depression, and decreased quality of life, are expected given the many hardships associated with the COVID-19 pandemic, a type of biological disaster (Osofsky et al., 2020; Hansel et al., 2022). It is urgent to use lessons learned from past disasters, such as Hurricane Katrina and the Deepwater Horizon (DWH) Gulf oil spill, to focus growth-based services following the many shared adversities due to the ongoing pandemic. Specifically, what are the mechanisms that help buffer negative mental health symptoms following prolonged adversity and build more positive longer-term outcomes, such as posttraumatic growth?

Hurricane Katrina in 2005 was noted as one of the worst natural disasters in US history (Knabb et al., 2006) and continues to be a marker of time for residents in Southeastern Louisiana. The disaster is considered both natural—due to the impacts of the hurricane—and technological or human caused—due to the breech in the levees. In addition to lives and property losses, residents were displaced, and cultural traditions and financial and emotional support systems were interrupted (Osofsky and Osofsky, 2021). The scale and long-term recovery provided ample opportunities to better understand how individuals respond to catastrophic events. Unsurprisingly, researchers found increased rates of suicide, depression, and posttraumatic stress disorder (Kessler et al., 2006; Osofsky et al., 2011b). Sequelae of symptoms and distress initiated or exacerbated by the disaster and subsequent recovery have continued for over a decade (Chan et al., 2015; Raker et al., 2019). Not only have preexisting conditions contributed to the longer-term challenges, but constant threat and response to additional disasters and the accumulation of adversities and stress are problematic for recovery (Hansel et al., 2015a).

Less than 5 years after the storm, many Hurricane Katrina survivors were impacted again by the DWH Gulf oil spill. The additional disruption to their income and social connectedness increased symptoms of anxiety and depression (Osofsky et al., 2011a), with many symptoms presenting as posttraumatic stress (Osofsky et al., 2017). The Gulf oil spill, a technical disaster, shared similar disruptions, economic constraints, and cultural disruptions as natural disasters. However, the marker for symptoms was heightened anxiety about their economic future, potential relocation for jobs, and health concerns from local water and seafood (Osofsky et al., 2011a; Sandifer et al., 2021). Mental health symptoms were heightened for over 2 years post-spill (Cope et al., 2013; Hansel et al., 2015b), in some cases due to other natural disasters (e.g., Hurricanes Harvey, Maria, Ida, etc.), personal crises, and community stressors. However, surveys also found that despite mental health challenges and losses, over 90% of participants reported being resilient or bouncing back from setbacks (Osofsky et al., 2011a; Hansel et al., 2020).

### Posttraumatic growth

Resilience, at the community level, can be measured by a community's ability to negotiate for and navigate toward resources (e.g., social, psychological, or physical; Ungar, 2011). Expanding the concept of resilience is the notion of posttraumatic growth (PTG). Characterizations of growth following adversity, stress, and trauma have been well documented for decades under a number of conceptual titles: (a) hardiness (Kobasa, 1979), (b) benefit finding (McMillen et al., 1997), (c) thriving (Abraído-Lanza et al., 1998), (d) stress-related growth (Park et al., 1996), (e) flourishing (Ryff and Singer, 1998), (f) adversarial growth (Linley and Joseph, 2004), and (g) posttraumatic growth (Tedeschi and Calhoun, 1996). PTG is thought to emerge as a result of coping with trauma rather than as a direct result of the trauma itself (Tedeschi and Calhoun, 1996). Given both the DWH Gulf oil spill and Katrina would have initiated coping with stress and adverse circumstances is warranted to expect growth in the wake of both events.

Overarchingly, the representations of growth describe an experience that exceeds resilience. Walsh (2002) aptly described growth as the experience of “bouncing forward” representing gains that emerge in the process of trauma adaptation. Tedeschi and Calhoun (1996) echo this idea in their definition of PTG. They describe PTG as positive psychological changes that result from coping with trauma (Tedeschi and Calhoun, 1996). They also developed the most widely validated measure of PTG called the posttraumatic growth inventory (PTGI; Tedeschi and Calhoun, 1996). The PTGI assesses growth in five realms: (a) appreciation of life, (b) relationships with others, (c) personal strength, (d) spirituality, and (e) new opportunities (Tedeschi and Calhoun, 1996; Taku et al., 2008).

### PTG controversies

Though PTG offers hope conceptually for positive adaptations to emerge in the face of trauma, the utility of PTG has garnered criticism. The controversies surrounding PTG highlight the challenges in distinguishing PTG from other adaptation trajectories and the resulting limitations in the practical utility of growth for trauma survivorship (Maercker and Zoellner, 2004; Wortman, 2004; Hobfoll et al., 2007; Saltzman et al., 2018).

These criticisms stem from the fact that the empirical evidence describing the relationship between growth and other trauma outcomes (e.g., distress) is inconclusive. To that end, some studies report that growth is a mechanism by which to reduce distress (Maercker and Zoellner, 2004; Wortman, 2004), while another found an inverse relationship between growth and distress (Kimhi et al., 2010), and still another reports no relationship at all (Eisma et al., 2019). Most recently, researchers have indicated the relationship reflects a curvilinear pattern with mild to moderate levels of distress positively associated with PTG, but higher levels inversely associated (Shakespeare-Finch and Lurie-Beck, 2014; Captari et al., 2021).

In answer to the controversies, more recent approaches to conceptualizing and measuring PTG have focused on the nuance regarding the *quality* of growth and changes in the quality of growth over time (Maercker and Zoellner, 2004; Zoellner and Maercker, 2006; Pat-Horenczyk et al., 2016; Saltzman et al., 2018) and behavioral representations of growth (Hobfoll et al., 2007). For example, Pat-Horenczyk et al. (2016) offered the first operationalization of the Janus face model of PTG proposed by Maercker and Zoellner (2004). In their study of breast cancer survivors, Pat-Horenczyk et al. (2016) paired the cognitive statement measured by the PTGI with coping style and distress symptomology to identify a multifaceted version of PTG. Expansion of PTG included representations of the illusory growth (i.e., a compensatory mechanism to reduce distress early in trauma recovery) and constructive growth (i.e., behavioral change that reflects the integration of growth over time) components proposed by Maercker and Zoellner (2004). Illusory and constructive growth have potential application in disaster recovery environments, but more studies are needed to quality of growth overtime.

### Purpose

Scholars have begun to focus on the nuances of PTG and changes in PTG over time, but the measurement and utility of this concept remain problematic. As such, continued studies are needed to understand the nuances of how PTG emerges in the lived experiences of disaster survivors and the ways in which PTG relates to other outcomes associated with trauma survivorship and cumulative adversities. The purpose of this study was to improve the understanding of the mechanisms that contribute to strengths following adversity in communities affected by two or more disasters. Specifically, we focused on residents that experienced both Hurricane Katrina and the DWH Gulf oil spill and their perceptions of personal, spiritual, or community changes in efforts to describe community resilience and posttraumatic growth. PTG and the strengths that help individuals persevere through challenging times are particularly important given the COVID-19 global pandemic and its ongoing toll.

##  Methods

Qualitative methodology was used to gain an in-depth understanding of strengths following adversity through the use of focus groups and interviews conducted in Lafourche, Jefferson, Orleans, Plaquemines, St. Bernard, and Terrebonne parishes. These sites were chosen based on existing relationships with the Department of Psychiatry and because of funding received to provide services and outreach to individuals affected by the DWH Gulf oil spill in these areas. Focus groups were conducted between June and September of 2015, approximately 10 years after Hurricane Katrina and 5 years after the oil spill. Each focus group lasted ~2.2 h (*M* = 132.4 min; minimum 85 min and maximum 185 min), and all participants received $25.00 for reimbursement of time. The study was approved by the institutional review board (IRB; amendment to #7540).

### Participants

Participants were recruited through a quantitative community survey (Osofsky et al., 2011a) and through community flyers. Participants provided an email address to receive reimbursement for participation in the quantitative survey—email addresses were not matched to data to ensure confidentiality of quantitative data, in the event courts demand release of protected data (Marshall, 1993). An email was sent to participants that were reimbursed for the quantitative survey, notifying them of the opportunity to participate in focus groups. Type of recruitment (community flyer or prior participation) was not collected. All participants were over the age of 18 years, and demographic data were not collected. The following southeastern Louisiana areas were represented: Bayou Dularge (seven participants), New Orleans East (6), Orleans Parish (10), Dularge (9), and St. Bernard (9), for a total of five focus groups and 41 participants. These five parishes (counties) were chosen based on their geographic location and impact of Hurricane Katrina; they were also the communities with highest percentages of claims following the Deepwater Horizon Gulf Oil Spill (Hansel et al., 2017). At a minimum, participants experienced both disasters either directly (i.e., personal loss, setbacks) or indirectly (i.e., future concerns, lack of recreation).

Each session was conducted by two facilitators, an faculty member and another faculty member, a postdoctoral fellow, or a research assistant. Both facilitators took handwritten notes, and a digital audio recorder was used. Names were not recorded next to individual responses, and identifying information was removed from the transcript. For consistency of responses, each focus group began with the same script and followed the same structure.

The focus group began by asking each participant to provide a brief overview of their disaster survival story, and the question was posed as, “how have you managed following past disasters or crises?” Given the geographical location, disaster was assumed to represent Hurricane Katrina, Hurricane Gustav, and the DWH Gulf oil spill. Three guiding strengths-based questions were then asked:

1. How are you able to adapt to change, bounce back, or keep going when things are really bad?
2. How has your community changed or grown despite the decade of disasters?
3. What advice/helpful information can you give others that incur many disasters?

The guiding questions were intentionally vague to ensure we were not leading or reinforcing existing individual constructs of PTG. Thus, we aligned the first question with resilience (Connor and Davidson, 2003) and the second and third questions with efforts to understand collective PTG (Black et al., 2022) and focused them on others and the community.

### Data analysis

Analysis was focused on the resilience of individuals affected by multiple disasters and at a minimum two (Hurricane Katrina and the Deepwater Horizon Gulf Oil Spill). Data were transcribed using Dragon Speech Recognition software. Given the variation in local dialects, at least two research assistants reviewed transcriptions. Five responses were either inaudible or removed due to side conversations unrelated to the focus groups. A total of 963 unique responses were analyzed and coded by a primary researcher—themes were confirmed by at least two other researchers and were determined based on percentages of responses and theoretical observations.

## Results

At the beginning of the focus group sessions, participants described their disaster experiences. They described the challenges of Hurricane Katrina and noted that only 5 years later, the DWH Gulf oil spill occurred. For some, it brought back unwanted memories. Others were still in the recovery process from Hurricane Katrina. All agreed the traumatic experiences, losses, and disasters have impacted their mental health (i.e., 326 of the responses described what they went through). In each focus group, participants noted some type of strength required to survive these experiences. The remaining responses (*n* = 637) were focused on contributing to or distracting from these strengths throughout recovery. The following themes were identified: connectedness (*n* = 259), coping (*n* = 94), spirituality (*n* = 60), adaptability (*n* = 47), and self-reliance (*n* = 23). Participants noted a growth mindset from the disasters and also acknowledged coinciding negative experiences associated with community change and loss (*n* = 154), where subthemes included change in connectedness (*n* = 97), crime (*n* = 26), and outsider (*n* = 31).

### Connectedness (*n* = 259)

The largest theme was connectedness which included concepts, such as community, family, friends, youth, generosity, and support. Personal and community supports received were particularly helpful to recovery; however, participants also noted giving to others as an important component of their wellbeing and sense of purpose.

Personal support systems were highly emphasized. One participant noted, “whether it's your own family or your church family or if it's just random neighbors. You pull closer together, everyone wants to help everyone else out.” Another noted cultural ties to get through, “as an African American community to celebrate our culture and celebrating your culture really helps you to recover.” Similarly, “that's the thing [support group] that really helps keeps you sane. Ya you'd rather rebuild within a community that you're familiar with than like starting over somewhere else.” Others noted the importance of younger family and how talking with children helped them. Participants noted, “talk to your children. Because they are going through stress too,” and, “you know I depended on them [children] like I treated them more like they were adults than like they were kids.”

Participants discussed helping others, either giving what they could or receiving from others. One participant noted,

[disaster] brings out the best and … worst in people. There is so many compassionate people out there. People would pay for our food; we would get up to leave and they would be like don't worry about it we got you … fortunately there was a lot of greater people out there that helped us.

One participant noted, “everybody takes care of their own stuff first, only natural, but if you still have water, you go and you help everybody else clean up and do whatever you can for each other.” Another noted the kindness of others, “if it wouldn't have been for them, I don't think I would have made it through, and it restores your faith in humanity.”

Community support was also helpful, “as a community I think everybody down here kind of looks out for each other.” However, some participants noted that the community connection was post-disaster, “I think before Katrina this community was just a place with a name I think after we became a community where we were neighbors organizing and fighting for a better health system or health or a better education system.” Another stated,

I know at least for me I was not involved in my community at all. You know I worked and then came home but now … I … get involved. So, I think that pulled up people that didn't focus on the community before.

### Coping (*n* = 94)

A predominant theme was coping, which included concepts such as adaptation, advocacy, positive change, and strength. Residents described the different ways in which they coped, including adaptation, humor, recognition of opportunity, strength, advocacy, and avoidance.

For some residents, adapting meant moving forward, “most forgive and forget.” For others, it did not, “you never forget it you just learn to adapt.” Some residents found that humor helped them adapt, so they tried to “find something to laugh about.” Humor helped to “move on to a brighter side and just keep the spirits up.” Many residents also tempered their sadness with recognition of new opportunities. One participant stated, “I feel like because of Hurricane Katrina I experienced what better education was.” Some expressed appreciation for what they felt was a new normal, such as, “it appears that New Orleans had a rebirth,” and, “really, New Orleans is the place to be for young tech students with the medical center and all that, the young professionals.”

Strength was a common theme in coping. For some residents, strength meant being strong for those in their immediate family; “I think for me it was mostly it was trying to be my mom's strength.” Some found strength in advocating for their communities; “so we all kinda like went to this training to teaching us how to do public speaking and what activists do and we shut down the landfill.” Another noted,

before I guess people were afraid to speak up but now, I notice our community, like if there's something that's not right, someone's gonna try to lead something and get it right and fight for what we need out here.

Some framed their strength as stubbornness; “most of us are very stubborn so that kind of probably helped us bounce back.” In true New Orleans fashion, some found expressive coping outlets stating, “that's why I dance.”

Although many talked about their positive coping skills, there was also a recognition of coping through avoidance; “I was always the one who hides my feeling, like I was sad or mad or worried, I would always just act like I'm strong but really I'm not.” Some experienced unsolicited avoidance on their behalf; “they told the kids they weren't allowed to bring up Hurricane Katrina to me.” One resident noted the perils of avoidance, “it's like you put it away in the back of your head and try to avoid it, but then you talk about it, and it all comes back to you like a ton of bricks.”

### Spirituality (*n* = 60)

Many participants noted the role that spirituality played when coping with the losses experienced during disaster. Given the location of the focus groups, this was most likely referring to Christian spiritual beliefs. Residents described different ways in which their higher power helped with recovery by providing strength, gratitude, and a church community.

Despite significant losses, one resident spoke about God stating, “where I come from, he's the star, he's the great, I can't say enough about him. I can't. That's how good he is.” According to another resident, “the Lord puts it there, and the Lord can take it away. He's always there for you.” Their unwavering faith in God was acknowledged by several residents. One resident commented, “we got a lot of faith … we have a lot of faith in God, and it don't pay to dwell on it.” Another resident stated, “it has to be God because nobody else can really help me as much as God does … because God can give you that strength that pushes people going.” One resident stated,

I think faith in God has a big role that plays in this community … a natural disaster, or any kind of disaster, will always look to God for help or guidance or … and I think that's what keeps this community unique and protected a lot of times.

Others recognized the importance of God and their church family and the role they played in supporting their families during the disaster. One resident noted, “but the good Lord was there helping us. The church helped us. That's how we managed.” Another resident recognized the support provided by their church: “the church families … they would send either financial support to the church, or food, clothing.” Local churches were supporting families, but church families from afar would also come and help families rebuild: “Churches from different states would call … they would get a group and from that point, they would come.” Several individuals noted that these individuals and groups “just wanted to serve God.”

Another common recognition was the ability to thank God for what they did have in their lives. One resident stated, “I really give the Lord all the thanks and the praise.” Another stated, “at the end of the day, you got to move on, thank God for your blessings, and be done with it.” Several residents recognized how God had provided them with a home. Other participants stated, “I thank God every day of my life that I still have a house,” and “we pretty much lost our home for the two hurricanes. God provided us with a new home.” Despite challenges during the disaster, they remained positive and continued to find strength in God, recognizing, “with the help of the Lord,” and “God knows best, he provided everything that we needed. He showed me the light at the end of the tunnel.”

### Adaptability (*n* = 47)

An important attribute recognized by residents was the role of resilience or adaptability in overcoming the challenges they faced. Types of adaptability included flexibility, using lessons learned, efforts to be part of the rebuilding, and personal change.

One resident stated his family managed through and took life as it came: “you can't control what happens in the world.” Another individual stated they “have to keep moving forward.” Part of moving forward was focusing on what could be controlled. For example, one resident stated, “do what you gotta do, if you can't get it done, something that can't be done, don't, just leave it and go do something you can do.” Other residents recognized the importance of controlling what they could and collaborated with one another to preserve the family atmosphere by “calling to coordinate their friends in all different areas to buy up houses in all the same areas so they keep that family atmosphere where they're friends are all still with them.”

Another resident recognized the importance of life lesson his father had taught them, stating, “can't sit and wait on everybody to come do for ya. You can't wait for all the handouts. And yes, people do need handouts and people do need help in time like this, [unintelligible]. But you got to help yourself.” Part of adaptability was being prepared as much as possible for disasters. One resident acknowledged the importance of their, “hurricane money,” set aside for hurricane season to support their families with emergency needs, such as gas and food. Residents also relied on their resiliency when they were placed in a position of fighting for the limited resources available. For example, one resident reported the challenges faced of going to Walmart as “there were so many people trying to get food and most of the time the shelfs were empty.”

Residents' desires to return and rebuild their community to “get back to normal” were recognized as important. Several residents also noted the cleanup process as a difficult time where adaptability was important. One resident recognized the difficulty in “throwing things away you never wanted to throw away,” while another resident noted, “you make a pile and you like, you gotta throw that way. Man, I don't want to.”

Residents also recognized the impact that positive personal changes had in their lives. After experiencing disasters, residents noted a new appreciation for life. Other residents recognized how fragile life could be. One resident recognized how his life had completely changed. He stated that prior to Katrina, “I was married, I had a new home, and I ran a business … during Katrina, my wife and I split, and she got the house, the business was destroyed.” Despite experiencing difficult times and life changing completely, the participant persevered and became “very optimistic about the future.”

### Self-reliance (*n* = 23)

Residents also recognized the role that self-reliance plays in facing disasters. Residents described the importance of taking care of oneself and the importance of autonomy and resourcefulness for disaster recovery.

One resident recognized the importance of relying on oneself to clean up and “don't put it off till other people does it for them.” Another resident stated, “don't sit down and wait for helps. Do as much as you can. Say your prayers first and try to salvage as much as you can. Try to lose as less as possible.” Another resident stated, “get it taken care of before it becomes a big problem cause the longer you wait the worse it is.” One last resident noted, “don't sit back … 90% of people down here actually do their own work they pick up and do” Residents recognized the importance of self-reliance and if they were to wait for help to arrive it may be too late to salvage their home and belongings. In addition, in times of disasters affecting large communities, it was common for family and friends to become separated and experience the same challenges, making it difficult to rely on others to rebuild. One resident stated, “well I remember after it happened like I had nobody you know. No friends all my family was all separated.”

Finding strength within oneself was noted by one resident stating, “you don't really have a choice. Things like this happen and you don't really have a choice but to move forward … forced to find your inner strength.” Residents recognize the importance of finding strength within themselves. One individual stated, “if you don't pull it together you are going to fall apart and you know you can't just go around and complain all the time.” In addition, residents were also focused on rebuilding their homes. With inner strength and perseverance, one resident stated, “we're just gonna go with it day by day and once they let us back in, we're gonna rebuild. There's no other place than home” Despite the challenges faced, residents found a common desire to find their inner strength and keep pushing toward building a strong future for themselves and their families.

### Change and loss negative (*n* = 154)

Participants acknowledged coinciding negative experiences associated with community change and loss (*n* = 154), throughout the recovery period. One participant stated, “I think a lot has changed. I mean you're riding around and notice how much is different.” This point was supported by head nods and elaborated through the subthemes, including change in connectedness (*n* = 97), increased crime (*n* = 26), and being outsider (*n* = 31).

#### Change in connectedness (n = 97)

Many participants reported experiencing changes in connectedness, especially in terms of neighborhood communities. Some participants in Orleans parish saw gentrification as a driver of change. One resident noted, “the impact of gentrification is forcing a lot of people who can't afford the new real estate values of the homes.” Another resident agreed, “the house for $25,000 before Katrina, now it's valued at over $200,000.” Some characterized the people who could afford the new real estate values as disconnected from the community. Although the expectation for newcomers to the community might be that they participate, “that they would actually try to start a community, something where we can get together and talk about the community together,” the reality was different: “they are not, how could I say, contributing to it. They are just flowing through it.” Another participant stated, “whatever happens in the community, they are just there.”

Participants noted that the areas being rebuilt were not necessarily close to them: “It's different now because everything now is further up and they're not really rebuilding much down here.” Some felt that economic reasons were driving the change. One participant noted,

There's still houses you see that have not been repaired or torn down and then you see empty blocks everywhere and that's really sad. But then a lot of people have come here to make money and stayed and more people I think left and that's kinda sad too.

Others felt the changes were happening for safety reasons; “they started building further up to escape the many hurricanes, the water problem we have here.” Some residents in outlying areas noticed an influx of New Orleans residents whose homes had not yet been rebuilt, and they felt that the community dynamics changed as a result; “a lot of the New Orleans people moved in this way … this was a totally different parish prior to Katrina and now it's so different.”

#### Crime (n = 26)

There was widespread agreement among residents that crime had increased following disasters. Theft was mentioned most often. One resident spoke of theft at their home: “another thing that made recovery hard too, like the house I am living in, they stole all the pipes.” Another talked about theft from the church: “they stole the pipes in our air conditioning system in the church three times.” As one resident summarized, “when we came back afterward down here it was like the Wild West.”

Explanations for the crimes varied. For some residents in New Orleans, it seemed to be a reprisal of previous behavior from established residents, such as, “they just got so comfortable back to their old ways that crime started getting worse and worse.” For some outside of New Orleans, it was brought in from other communities by displaced individuals. One participant stated, “you have other populations from different places that have come into this area that have brought crime have brought stuff that was never here before.” Others found their neighborhoods more peaceful than before: “it's quiet. They didn't have a lot of people around. The crime rate wasn't as high as a lot of areas.”

#### Outsider (n = 31)

The new environments that displaced residents had to navigate brought their own challenges. Many participants described feeling like an outsider while they were evacuated, mostly due to the amount of attention they received. With the attention came a certain amount of celebrity; “anywhere we were, every school we went to, we felt like all eyes on us. We were the celebrities, like these are the Hurricane Katrina victims.” There were mixed feelings about offers of support: “they're trying to talk to you but sometimes it as too much. And to me it was like I just wish people just backed off a little bit.” This situation was difficult to navigate for some, noting, “I don't know exactly where that boundary line is to where you're helpful and you're being too much.” Another stated, “it was just hard for me to meet new friends and stuff all the time like every month I would switch to a different school.” Many felt labeled:

so, I had to go over to a huge school. And then they just labeled you as a refugee or a victim and I'm just like okay you guys can't label … how is that gonna make me feel better?

Some also felt they had become the objects of pity. One resident said, “they played this sad song and I'm just like, “oh my God, can you stop feeling sorry for me.”” Another resident agreed, “it's like “we wanna be your friends cuz we feel sorry for you” and it's like, “Great, um, I don't wanna be your friend.””

## Discussion

The underlying mechanisms contributing to posttraumatic growth are debated and have limited application to disaster-specific traumas, particularly in communities impacted by cyclical or cumulative disasters. Similarly, PTG as a concept has posed challenges in measurement at the individual level and even greater challenges in translating the assessment of PTG to the community level in the context of collective trauma (Black et al., 2022). Though few studies have explored the emergence of PTG following community-level trauma (e.g., Strasshofer et al., 2018; Lee et al., 2019; Waters et al., 2021), none have developed a specific community-level tool for measuring PTG. At the individual level, Rhodes and Tran (2013) highlighted items useful for community insight following Hurricane Katrina, which include closeness and faith in others. This study aimed to improve the understanding of strengths following adversity in communities affected by both Hurricane Katrina and the DWH oil spill focusing on survivor perceptions of personal, spiritual, or community changes in efforts to describe community resilience and posttraumatic growth. These findings also provide insight into collective representations of PTG. Findings revealed five themes: connectedness, coping, spirituality, adaptability, and negative experiences. Themes are discussed in the context of PTG realms of appreciation of life, relationships with others, personal strength, spirituality, and new opportunities (Taku et al., 2008).

### PTG—Relationship with others

Perhaps the most salient with our study is the theme of connectedness that aligns with the posttraumatic growth realm of relationship with others. Connectedness with others and communities seems more appropriate in the disaster context of PTG, given the link with social capital. Researchers propose that social capital following a disaster can help us to gain a better understanding of recovery (Ritchie and Gill, 2007). Further, maintained or increased social capital has been found to be associated with an improvement in the recovery trajectory, and low or reduced levels of social capital have been associated with less desirable recovery outcomes (Marín et al., 2015). One participant noted,

They say oh, you live at the end of the world. I'm like, no, this is where it begins at. But you know, like a lot of them, a lot of people, they ask you, how can y'all live down there knowing they gon have hurricanes? I wouldn't want to live anywhere else. Everything you grew up with here, the community, it's like you're not going through this alone, you're sharing this tragedy with your friends and families.

Similarly, but in a different population, support and cohesion have been found to mitigate increases in avoidant coping after combat deployment for military personnel (McAndrew et al., 2017). In advice to future disaster survivors, one participant instructed to “gather your support group because you're going to need that support group.” This thematic grouping of connectedness or relationship to others further illustrates the narratives of participants who found strength within their communities. Social capital has also been found to be an individual-level protective factor following the Earthquake and Tsunami in Japan (Hikichi et al., 2020), further suggesting the need for community-level indicators in PTG inventories.

### PTG—Personal strength

Perceived changes in self (adaptability, personal change, and self-reliance) align with the category of personal strength on the PTGI (Tedeschi and Calhoun, 1996). Participants framed strength in terms of personal and community strength. In terms of personal strength, one participant said sometimes adversity can help you to “realize how strong you really are.” One participant said of their community, “When somebody's down I think everybody tries to pick everybody up or just sometimes a helping hand or a helping word, it gives everybody strength.” This community strength sometimes translated to advocacy, as was the case when several members of a community “went to this training to teach us how to do public speaking and what activists do” when fighting against a toxic landfill in their neighborhood. Yosso and Solórzano (2005) defined resistant capital as “those knowledges and skills fostered through oppositional behavior that challenges inequality” (p. 80). Chioneso et al. (2020) proposed a framework of community healing that recognizes justice as both a condition and an outcome of healing and adaptability.

As one participant explained regarding adaptability, “We have to move forward, we have rebuilt and pick up where we left off … we have to have some fun to it, we have to have fight to it.” Researchers believe that adaptability can have positive effects on mental health and physical health. Buckingham-Howes et al. (2017) found people who had scored higher in adaptability on the Connor–Davidson Resilience Scale had higher overall health and physiological qualities of life 4.5 years after the Deepwater Horizon oil spill. However, both female identity and resource loss have been noted as risk factors for resilience post-spill (Lightfoot et al., 2020). Bos et al. (2016) found that cultivating personal strength, including sense of humor, helped people to preserve wellbeing despite the presence of symptoms of depression, anxiety, and stress. Participants who “would crack jokes about the hurricane” used humor to facilitate adaptation. Humor has been found to be independently and positively associated with resilience following multiple disasters (Cherry et al., 2018). Collective findings from this study highlight the role of adaptability as a mechanism for resilience (Abramson et al., 2015). Adaptability as growth allows for the acceptance of change or the *new normal* in collective disaster recovery.

### PTG—New opportunities and appreciation of life

The PTG realms of new opportunities and appreciation of life seem to be the most misaligned with findings from the current study. Studies following Hurricane Katrina have noted the importance of new priorities (Rhodes and Tran, 2013) in the facilitation of PTG, yet this was not a theme for the current study. It is possible that in a cumulative disaster context, it is very difficult to consciously apply these concepts when in constant disaster recovery mode, and impacts to the physical environment are reminders of negative change. In part, this may reflect the changes in the PTG that emerge over time. Perhaps appreciation of life and new opportunities requires a greater period of healing posttrauma—often not afforded to individuals living in disaster-prone regions.

Further, in a disaster context, focusing on strengths should not disguise the many negative experiences. Rather community change and loss were a major finding in the current study, where subthemes included change in connectedness, crime, and feeling as an outsider. The reality of co-occurring negative sequelae and PTG has been documented in other trauma typologies (e.g., work with breast cancer survivors and veterans identify a profile of adaptation referred to as “struggling growth” in which participants endorse high levels of psychological distress and PTG simultaneously; Pat-Horenczyk et al., 2016; Saltzman et al., 2018). Struggling growth is thought to be a transitory profile in which some survivors resolve distress symptomology, a process that similarly requires time between trauma exposures that may not be afforded to disaster-prone communities.

Participants described the community change and loss associated with their new normal. Some family and community members were permanently displaced. Neighborhoods changed with gentrification and increased crime. Finally, being considered an outsider or victim of the disaster added to distress. However, a few participants talked about positive change and feeling that New Orleans “really is the place to be now.” Tedeschi and Calhoun (1996) view positive change as a key component of posttraumatic growth, while Zoellner and Maercker (2006) caution that researchers should be aware of the potential for survivors to inflate positive outcomes. As mentioned in the results, many participants felt that the post-disaster changes had been largely negative.

#### Negative change and loss

Much in the same way community connectedness was important to participants, community change was also important in shaping their PTG. Participants acknowledged coinciding negative experiences or reduced social capital as part of the recovery. In the context of PTG, it is important to not gloss over the negative elements of the disastrous event in that they are an illusory coping mechanism and an important part of the recovery process. Recognition of the negative elements also addresses the criticism that PTG *gold plates* the process of coping (Zoellner and Maercker, 2006) and represented the lived experiences of study participants. Participants discussed the negative changes they had experienced in their communities and sense of connectedness, in post-disaster crime rates, and in being treated like or feeling like outsiders in their evacuation locations, offering an important collective element that may influence experiences of PTG following collective trauma and disaster.

Participants who framed negative changes in their communities as gentrification were observing a documented phenomenon. Between 2000 and 2015, areas of New Orleans that were less prone to flooding saw significant increases in White, educated, higher-income residents and decreases in Black residents with lower levels of education and income (Aune et al., 2020). As the increased home prices were “forcing them out,” residents found their predisaster communities dispersed. Morris and Deterding (2016) found a positive association between the dispersion of social networks and the likelihood of posttraumatic stress following Hurricane Katrina.

Some residents spoke of directly experiencing crime, such as the theft of pipes from their home or their church. Others spoke of hearing of increasing crime rates, especially in New Orleans East, one of the areas that experienced some of the worst flooding after Hurricane Katrina. Though some scholars believe disaster-related crime threatens to undermine authority and should be harshly dealt with (Kuo et al., 2012), others see looting as a response to inequality and marginalization and a desperate attempt to voice grievances to a system that would not otherwise hear them (Van Brown, 2019). Noting that several news outlets relied on fictitious rumors of post-disaster looting to boost ratings, Berger (2009) argued the media had a hand in exacerbating the incarceration crisis. However, as the firsthand experiences of some participants indicate, there was, indeed, some post-disaster crime. Curtis and Mills (2011) supported the speculation of participants who heard that crime was worse in more heavily affected areas when they found that crime was inversely related to the amount of activity on recovering streets in the Holy Cross neighborhood of New Orleans.

Many participants who evacuated reported being treated like outsiders by members of their host community. They often felt negatively labeled, sometimes as “refugees” and sometimes simply in terms of race and class. A phenomenological study in San Antonio concluded that evacuee adaptation was affected by such elements as government actions, media portrayals, and race relations, all of which were connected to the refugee label (Kristjánsdóttir and DeTurk, 2013). One study in Houston confirmed that negative attitudes toward evacuees from Hurricane Katrina were correlated with negative attitudes toward immigrants and that these negative attitudes increased over time (Shelton and Coleman, 2009). While another Houston study found similar results, the strength of antagonistic attitudes decreased—yet persisted—over time (Warren, 2013). Similarly following the oil spill, community connectedness was not a protective factor for everyone. Individuals in the fishing industry reported higher depression and social support (Parks et al., 2020), suggesting additional considerations must be given to type and complexities within connectedness and the role reduced social capital plays in recovery and resilience.

### PTG—Spirituality

Spirituality was another important theme in the study and may represent a cultural nuance and importance of religion in the study location—Southeastern Louisiana. One participant noted that “a lot of the stuff I was saying was just negative, but I was just saying because of the death … that I experienced, but you know trying to stay positive and looking at the goodness of God.” However, many have noted the meaning making or sense of purpose derived from the trauma rather than directly tied to a higher power (Park et al., 2012). Other studies following the DWH Gulf oil spill suggested mixed effects with low religious individuals in highly religious communities displaying negative coping, such as problematic drinking (Drakeford et al., 2020). Further, spirituality is not a dichotomous measure, and the various degrees of religiosity or spirituality may be less protective and increase vulnerability to poor mental health, especially anxiety (Stroope et al., 2020). For natural and technological disasters, the meaning making is not event specific or tied to a higher power but around the changes to the community and one's spirituality as a lens to view those changes (Rhodes and Tran, 2013). Thus, for disasters and the current study, God and sense of purpose to the community or connection to place contributed strengths following adversity. Yet, additional studies are needed to fully understand spirituality and PTG.

### PTG—Controversies

Perhaps the most apparent controversy toward PTG that aligns with the current study's findings is the concept of constructive growth (Maercker and Zoellner, 2004) and the behavioral changes that reflect the integration of growth over time (Hobfoll et al., 2007; Saltzman et al., 2018). The current findings highlight the challenges faced in disaster-prone communities that may not have time between disasters to improve the quality of growth they experience. In our data, the development of positive coping toward future disasters was evident in the emphasis on coping noted by responses. Participants found different ways of coping with disasters. Some talked about adapting to their new realities, some talked about finding their strength, some related positive changes that had occurred in the aftermath, and some relied, at least temporarily, on maladaptive coping techniques, such as avoidance and hiding their feelings. Similarly, scholars have postulated that avoidant coping may facilitate PTG, which would demonstrate the illusory aspect of the Janus face model (Zoellner and Maercker, 2006; London et al., 2020). However, avoidant coping has also been found to have a strong association with higher posttraumatic stress and depressive symptoms (Bistricky et al., 2019). Though coping may overlap with personal strengths, it is important to note that improved coping or awareness of coping is critical to disaster recovery, especially in regions consistently exposed to disasters.

## Limitations and future research

Though the findings shed light on the context of posttraumatic growth in the context of multiple disasters, they are not without limitations. The lack of generalizability due to the qualitative nature of the study limits applicability to other disasters and communities. Thus, we cannot extend results to individuals beyond the focus group participants; however, their experience provides potential insight into posttraumatic growth and warrants future research. Similar to the study's design, focus group methodology may have biased participant responses, especially on community connectedness. Similarly, individuals experiencing PTG may have been more likely to self-select for participation. Given the ongoing litigious nature of the oil spill, we did not collect any sociodemographic data to increase anonymity; however, this also represents a major limitation for replication. Timing since the disaster and differing types of disasters may have also influenced the results. Though posttraumatic stress disorder and posttraumatic growth are dependent on prolonged time from the trauma, it is possible that threats to internal validity, such as history and maturation, may be conflating results specific to hurricanes and the oil spill. Further, we did not assess the perceived significance of loss and trauma associated with each disaster, nor did we ask participants to define what community meant to them. Additional research is needed to understand the meaning making behind loss and community context in disaster recovery. Given the location of the focus groups, we assumed spirituality referenced Christian values; however, other religions and spiritual practices, such as meditation, may also be beneficial and should be explored. Future research should test these findings in other communities and with other types of disasters. Quantitative methodologies would also be beneficial to increasing numbers and understanding posttraumatic growth in the context of other community disasters.

## Conclusion

In the immediate aftermath of disasters, the devastating effects make it difficult to view posttraumatic growth as a possible outcome. Cumulative disasters or disasters with ongoing recovery may be particularly challenging. The field of posttraumatic growth demonstrates evidence in disasters; however, much of the literature has focused on individual rather than collective growth (Wlodarczyk et al., 2017; Ali et al., 2021). The purpose of the current study was to improve the understanding of collective growth and the strengths following adversity in communities affected by both Hurricane Katrina and the DWH Gulf oil spill. Our findings align with the larger PTG literature and highlight the importance of community connectedness. Commitment to and support from the community were noted consistently in all themes and should be considered as an additional realm in PTG, especially for disasters. Despite acknowledgments of negative experiences from the disaster and changes to the community, participants did not waiver in their commitment to the community. Results suggest that efforts to identify and build community connectedness prior to disasters and emphasized throughout recovery are critical to rebuilding and individual and collective coping. The importance of community support and social capital is considered as a realm in PTG following collective traumas, such as disasters, and explored further on how community connectedness can be supportive in the aftermath of the COVID-19 pandemic.

## Data availability statement

The original contributions presented in the study are included in the article/supplementary files, further inquiries can be directed to the corresponding author.

## Ethics statement

The studies involving human participants were reviewed and approved by Louisiana State University Health Sciences Center. The patients/participants provided their written informed consent to participate in this study.

## Author contributions

Study conception and design: HO, JO, and TH. Data collection: TH. All authors contributed to the analysis and interpretation of results, draft manuscript preparation and reviewed the results, and approved the final version of the manuscript.

## Funding

The Gulf Region Health Outreach Program was developed jointly by BP and the Plaintiffs' Steering Committee as part of the Deepwater Horizon Medical Benefits Class Action Settlement, which was approved by the U.S. District Court in New Orleans on January 11, 2013, and became effective on February12, 2014. The Outreach Program is supervised by the court and is funded with $105 million from the Medical Settlement.

## Conflict of interest

The authors declare that the research was conducted in the absence of any commercial or financial relationships that could be construed as a potential conflict of interest.

## Publisher's note

All claims expressed in this article are solely those of the authors and do not necessarily represent those of their affiliated organizations, or those of the publisher, the editors and the reviewers. Any product that may be evaluated in this article, or claim that may be made by its manufacturer, is not guaranteed or endorsed by the publisher.
